# Long Non-Coding RNA Expression in Alpha-1 Antitrypsin Deficient Monocytes Pre- and Post-AAT Augmentation Therapy

**DOI:** 10.3390/ncrna9010006

**Published:** 2023-01-10

**Authors:** Stephen G. J. Smith, Catherine M. Greene

**Affiliations:** 1 Department of Clinical Microbiology, Trinity College Dublin, St. James’s Hospital, Dublin 2, Ireland; 2 Lung Biology Group, Department of Clinical Microbiology, RCSI University of Medicine and Health Sciences, Education and Research Centre, Beaumont Hospital, Dublin 9, Ireland

**Keywords:** deficiency, augmentation therapy

## Abstract

Long non-coding RNAs (lncRNAs) regulate gene expression. Their expression in alpha-1 antitrypsin (AAT) deficiency has not been investigated. Treatment of AAT deficiency involves infusion of plasma-purified AAT and this augmentation therapy has previously been shown to alter microRNA expression in monocytes of AAT-deficient (ZZ) individuals. Here, we assess the effect of AAT augmentation therapy on the lncRNA expression profile in ZZ monocytes. Peripheral blood monocytes were isolated from ZZ individuals pre (Day 0)- and post (Day 2)-AAT augmentation therapy. Arraystar lncRNA microarray profiling was performed; a total of 17,761 lncRNAs were detectable across all samples. The array identified 7509 lncRNAs with differential expression post-augmentation therapy, 3084 were increased and 4425 were decreased (fold change ≥ 2). Expression of many of these lncRNAs were similarly altered in ZZ monocytes treated ex vivo with 27.5 μM AAT for 4 h. These properties may contribute to the manifold effects of AAT augmentation therapy.

## 1. Introduction

Alpha-1 antitrypsin (AAT) is a serine proteinase inhibitor encoded by the *SERPINA1* gene [1]. It is expressed primarily by hepatocytes but also by neutrophils, monocytes and airway epithelial cells and its primary target is neutrophil elastase; however, it can also inhibit other proteases. Furthermore, AAT possesses an array of immuno-modulatory and anti-inflammatory properties (recently reviewed in [2]).

Over 200 *SERPINA1* alleles have been described. The normal variant is referred to as ”M”. In AAT deficiency, wherein an individual inherits two mutant alleles, the most frequent and severe disease-associated mutation is the ”Z” allele, which leads to expression of aberrantly folded AAT proteins within hepatocytes. This leads to low levels of AAT in the circulation and an inadequate antiprotease protective screen in the lungs and was first recognized in 1963 by Laurell and Eriksson [3]. The hallmark of AAT deficiency-related lung disease is neutrophil-dominated inflammation resulting in emphysema.

Treatment of the lung manifestations of AAT deficiency includes many standard therapies for chronic obstructive pulmonary disease (COPD) in addition to ”augmentation therapy” with human plasma-derived, purified AAT. AAT augmentation therapy aims to restore AAT levels in the circulation to that of an MM individual, i.e., a “putative protective threshold” of 11 µM (0.57 g·L^−1^) AAT in serum [4]. The benefits of augmentation therapy in AAT-deficient patients are well-established, and AAT augmentation therapy is a safe and well-tolerated therapy [5]. It significantly reduces loss of lung density and interferes with disease progression in patients with AAT deficiency-related emphysema [6,7]. AAT augmentation therapy also reduces proinflammatory immune processes [2,8,9,10].

Previously, we reported that microRNA expression in ZZ monocytes is altered in response to treatment with AAT both in vivo and ex vivo [11]. In particular, we linked this observation to AAT-mediated inhibition of NFkappaB-regulated microRNAs, and proposed that these effects may contribute to the anti-inflammatory effects of AAT augmentation therapy. Long non-coding RNAs (lncRNAs), similar to microRNAs, are a large class of non-coding RNAs that can regulate gene expression [12]. They are potentially implicated in all aspects of human biology. Unlike miRNAs, lncRNAs have various modes of action due to their ability to interact with RNA, proteins and DNA, and their capacity to act both in cis and in trans. Given that altered miRNA expression could be observed in ZZ monocytes in response to AAT augmentation, here we explore whether lncRNAs might be similarly affected.

## 2. Results

### 2.1. LncRNA Expression Profile of ZZ Monocytes

Monocytes were isolated from n = 5 ZZ AAT-deficient individuals (3 males, 2 females, mean age 49.91 ± 3.87 years) and the lncRNA expression profile was measured by array. Of the 30,586 lncRNA probes on the microarray, a total of 17,761 lncRNAs were detected in the ZZ monocytes.

### 2.2. Effect of AAT Augmentation Therapy on lncRNA Profile

**Figure 1:**
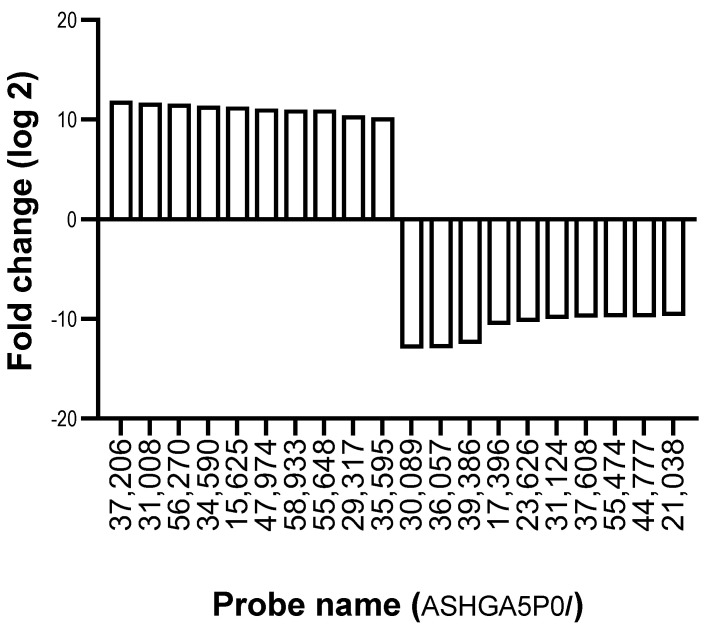
Ten most highly up- and downregulated lncRNAs in monocytes of AAT-deficient patients receiving AAT therapy versus monocytes of AAT-deficient patients not receiving AAT therapy.

**Figure 2:**
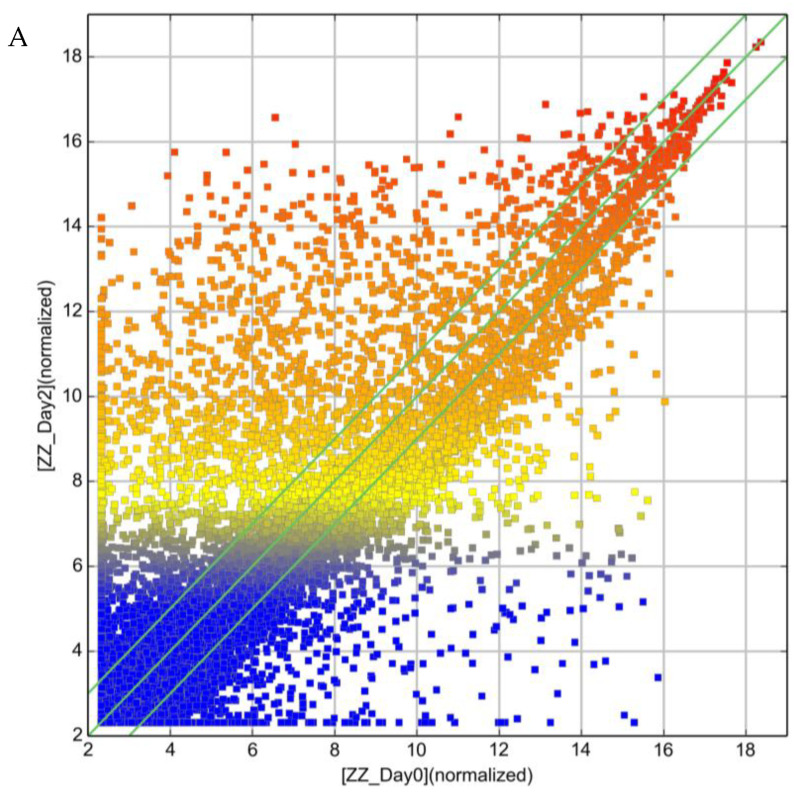

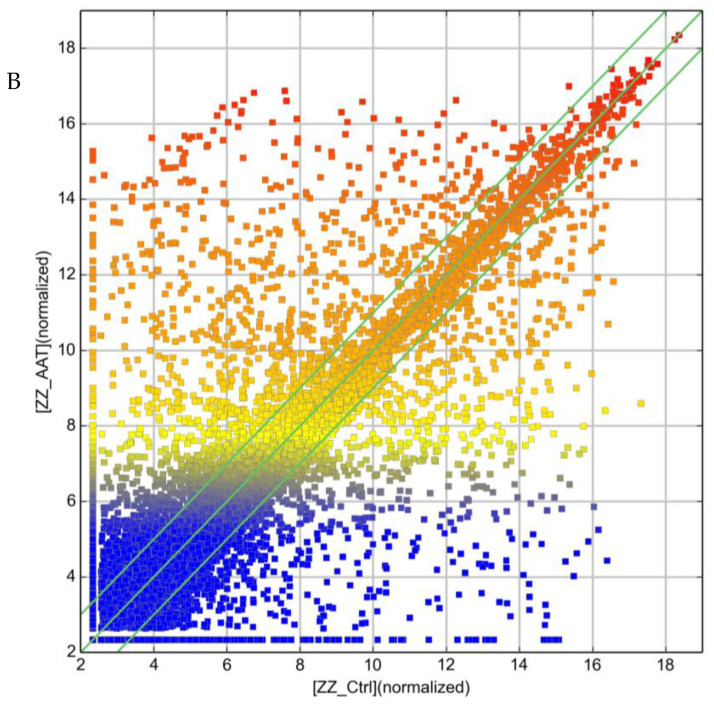
Scatter plots of lncRNA expression variation between (**A**) ZZ monocytes isolated 48 h post-AAT augmentation therapy (Day 2, Y axis) array and ZZ monocytes from untreated individuals (Day 0, X axis), and (**B**) ZZ monocytes treated ex vivo with 27.5 μM AAT for 4 h (ZZ_AAT, Y axis) and untreated control ZZ monocytes (ZZ_Ctrl, X axis). The outer green lines represent ±2.0 fold difference between the two compared samples.

The lncRNA expression profile of ZZ monocytes isolated 48 h post-AAT augmentation therapy (herein referred to as ”Day 2”) was also measured by array and compared to ZZ monocytes from untreated individuals (Day 0). Figure 1 and Table 1 show the top 10 lncRNA increases and decreases in response to augmentation therapy. In total, 7509 lncRNA probes were found to have a fold-change ≥2.0 (equal to a log_2_ FC of 1.0)-altered expression in ZZ monocytes post-augmentation therapy versus Day 0; 3084 were increased and 4425 were decreased (Figure 2A, Heat map provided as Supplementary Figure S1A). ENST00000430815 was upregulated to the greatest extent, with a log_2_ FC of 11.9, while ENST00000559861 was downregulated to the greatest extent, with a log_2_ FC of −12.9.

### 2.3. Ex Vivo Treatment of ZZ Monocytes Alters lncRNA Expression

**Figure 3:**
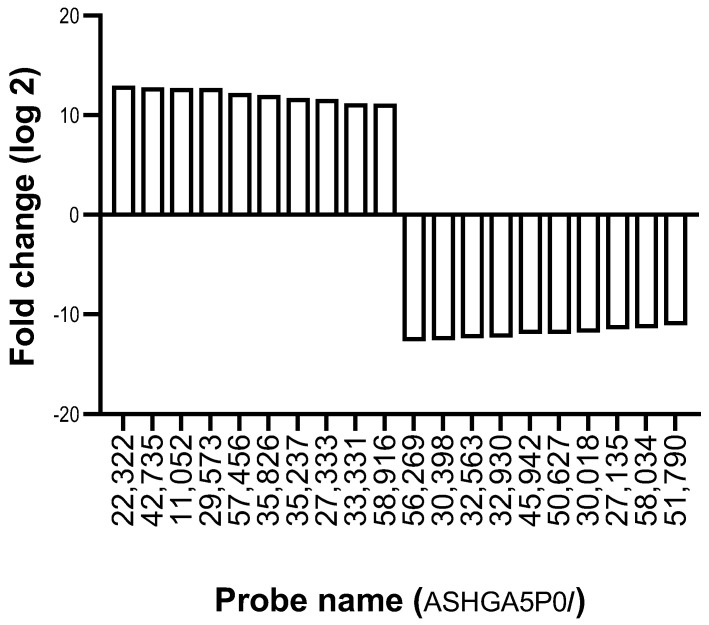
Ten most highly up- and downregulated lncRNAs in AAT-deficient monocytes treated ex vivo with AAT versus those not treated ex vivo with AAT.

Next, the lncRNA expression of ZZ monocytes treated ex vivo with 27.5 μM AAT for 4 h was profiled. The selection of 27.5 μM AAT for the ex vivo studies was chosen as a translationally relevant concentration based on the post-augmentation serum level of the ZZ patients measured on Day 2 (30.3 ± 4.7 μM, compared to pre-augmentation therapy AAT levels of 8.1 ± 0.8 μM). Figure 3 and Table 2 show the top 10 lncRNA increases and decreases in ZZ monocytes treated for 4 h with AAT versus untreated control cells. In these samples, ENST00000554529 was upregulated to the greatest extent, with a log_2_ FC of 12.95, while TCONS_00001565 was downregulated to the greatest extent, with a log2 FC of −12.7.

In total, 5148 lncRNA probes were found to have a fold-change ≥ 2.0-altered expression in ZZ monocytes treated ex vivo with AAT versus untreated cells; 2587 were increased and 2561 were decreased (Figure 2B, Heat map provided as Supplementary Figure S1B).

### 2.4. Comparison of In Vivo and Ex Vivo Effects of AAT on ZZ Monocytes

As it was not possible to validate the array results by qRT-PCR in the original samples or additional samples due to insufficient sample volumes, we instead undertook an alternative validation process whereby we assessed whether the 10 lncRNAs most highly altered by augmentation therapy in vivo were similarly altered, i.e., increased or decreased, in ZZ monocytes treated ex vivo with 27.5 μM AAT for 4 h, this concentration of AAT being equivalent to the in vivo augmentation therapy dose. Of the top 10 up- and downregulated lncRNAs in vivo, only 3 upregulated lncRNAs and none of the downregulated lncRNAs were similarly altered in ZZ monocytes treated ex vivo with AAT (Table 3). 

As the corollary to this, we also assessed whether the top 10 lncRNAs altered after 4 h ex vivo treatment were altered in ZZ patients receiving augmentation therapy. Of the top 10 up- and downregulated lncRNAs ex vivo, 5 upregulated lncRNAs and none of the downregulated lncRNAs were similarly altered in ZZ monocytes receiving augmentation therapy on Day 2 (Table 4).

**Figure 4:**
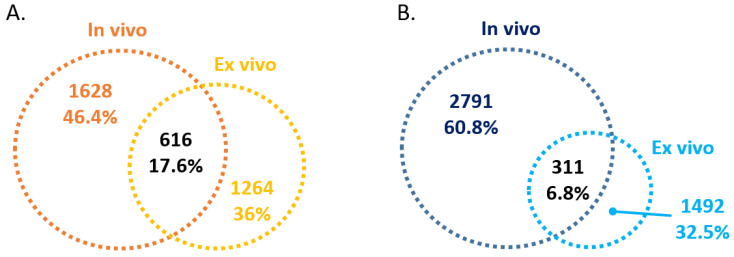
Venn diagram of in vivo and ex vivo differentially expressed lncRNAs. (**A**) Upregulated and (**B**) downregulated lncRNAs in ZZ monocytes isolated 48 h post-AAT augmentation therapy (In vivo) and ZZ monocytes treated ex vivo with 27.5 μM AAT for 4 h (Ex vivo).

We also assessed what percentage of the differentially expressed lncRNAs were similarly altered in the entire dataset by cross-comparing the list of lncRNAs altered in the ex vivo AAT-treated ZZ monocytes and in the ZZ monocytes receiving AAT augmentation therapy in vivo (i.e., Day 2). After removal of duplicate or multiple gene probes, 17.6% (n = 616) of the increased lncRNAs (Figure 4A) and 6.8% (n = 311) of the decreased lncRNAs (Figure 4B) displayed a similar expression pattern in both sample sets. These lncRNAs are listed in Supplementary Tables S1 and S2. Full data on all differentially expressed lncRNAs are available in Supplementary Dataset S1.

### 2.5. Pathway Analysis

**Figure 5:**
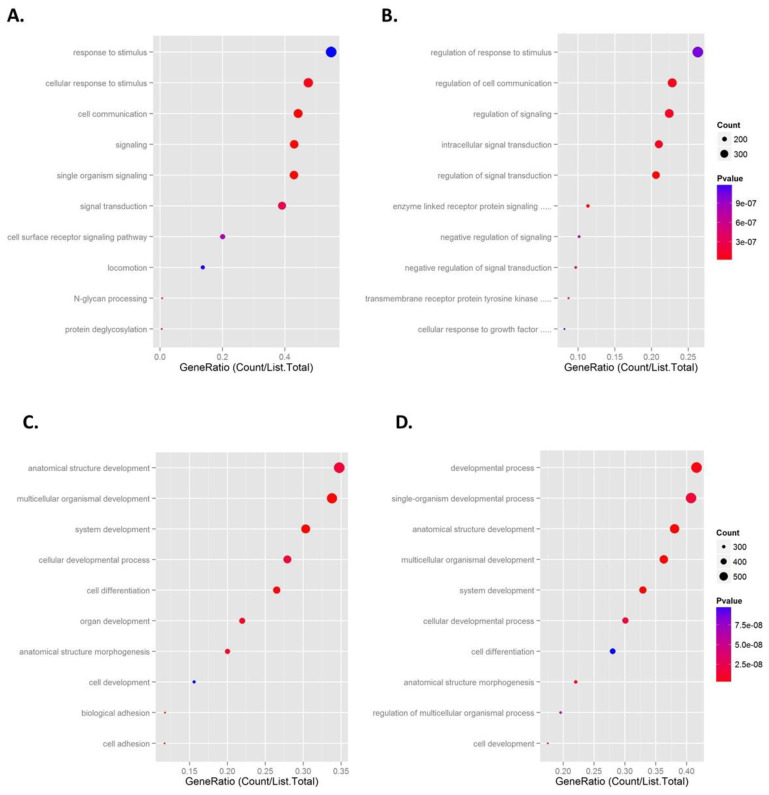
Dot plots of the gene ratio values of the top 10 pathway identifiers used in KEGG. (**A**,**B**) Upregulated and (**C**,**D**) downregulated pathways in ZZ monocytes isolated 48 h post-AAT augmentation therapy (**A**,**C**) and ZZ monocytes treated ex vivo with 27.5 μM AAT for 4 h (**B**,**D**).

As previously reported, 334 genes were commonly downregulated by AAT [11]. There were 567 genes upregulated in the Day 2 versus Day 0 samples and in the ZZ monocytes treated with AAT versus those untreated. Pathway analysis of these differentially expressed regulated mRNAs was performed. Figure 5 shows dot plots of the gene ratio value of the top ten most significant enrichment pathways for the upregulated (Figure 5A,B) and downregulated (Figure 5C,D) mRNAs in the in vivo and ex vivo samples. For the upregulated mRNAs, there was little overlap between the enrichment pathways in both sets of samples. However, as previously reported, for the downregulated genes, seven of the top ten enrichment pathways in the ZZ monocytes receiving AAT augmentation therapy were also represented in the top ten pathways in ZZ monocytes treated with 27 μM AAT for 4 h [11]. These pathways are specifically related to developmental and morphogenesis processes and cell development and differentiation.

## 3. Discussion

This small observational study provides a snapshot into the expression profile of lncRNAs in peripheral blood monocytes of AAT-deficient individuals, and the effect of AAT augmentation therapy on this profile. A total of 17,761 lncRNAs were detectable across all samples. A total of 7509 post-augmentation therapy lncRNAs displayed differential expression; 3084 were increased and 4425 were decreased. Many of these lncRNAs were similarly altered in ZZ monocytes treated ex vivo with AAT. These properties may contribute to the manifold effects of AAT augmentation therapy [2,6,7,8,9,10,11].

Disappointingly, no function has yet been assigned to the four most highly up- or downregulated lncRNAs identified in vivo or ex vivo, although some details are available. The gene symbol for the sequence name ENST00000430815 is C21orf91 overlapping transcript 1 (C21orf91-OT1) and is encoded on chromosome 21; according to Genotype Tissue Expression (GTEx) release version 6, its highest median expression is in testis with the next highest expression in whole blood. ENST00000559861 is an antisense lncRNA encoded on chromosome 15. The gene symbol for ENST00000554529 is RP11-14J7.6 and is of unknown function, whilst TCONS_00001565 encodes an intergenic lncRNA (lincRNA) on chromosome 1. 

Of greater interest are the lncRNAs that are differentially expressed in both datasets. Only eight of the top forty up- or downregulated lncRNAs were detected when both datasets were cross-compared; all were upregulated. Of these, the most interesting in the context of this study is ENST00000439606, which is the lncRNA solute carrier family 8 member A1 antisense RNA 1 (SLC8A1-AS1). This lncRNA targets the sodium calcium exchanger, SLC8A1, and its upregulation has been shown to increase interleukin 10 (IL-10) and decrease IL-1β, IL-6, transforming growth factor α, nitric oxide, inducible nitric oxide synthase and endothelial nitric oxide synthase in a mouse model of myocardial infarction [13]. In monocytes, SLC8A1, also known as NCX1, plays a critical role in high salt-triggered sodium ion influx and calcium ion efflux [14]. SLC8A1/NCX1 is required for amplifying inflammatory and antimicrobial macrophage responses following high salt exposure and inhibiting its activity impairs high salt-induced inflammatory signaling, infection-triggered autolysosome formation and antibacterial activity. mRNA expression in ZZ monocytes receiving augmentation therapy or treated ex vivo with AAT has been previously reported [11]. Therein, SLCA1/NCX1 expression was decreased −1.46 log_2_ FC in Day 2 samples, but not in ZZ_AAT vs. Ctrl samples (1.088 log_2_ FC). Further studies could determine whether AAT is directly affecting this pathway in ZZ monocytes and what the actual consequences are for monocyte function.

Comparison of the entire dataset revealed 616 upregulated and 311 downregulated lncRNAs in both sample sets. These lncRNAs are represented across all subgroup classes of lncRNAs: enhancer LncRNAs, lincRNAs, human homeobox transcription factors (HOX) cluster lncRNAs, lincRNAs nearby coding genes, and enhancer lncRNAs nearby coding genes (both distances < 300 kb). Altered expression of some of these lncRNAs may contribute to the manifold effect of AAT augmentation therapy. Validation studies using additional cohorts and in vitro mechanistic studies will help to elucidate the roles of these lncRNAs and their consequences for AAT-deficient individuals receiving augmentation therapy. Given that the ex vivo treatment was for 4 h whereas the in vivo effect was measured after 2 days, it is not surprising that there are differences between the two datasets. Whilst the lncRNAs that are altered in both datasets are of strong interest, those that are altered in only one of the datasets and not the other are of equal interest and should be considered in the context of temporal, paracrine and/or solely monocyte-specific effects, amongst others.

Disappointingly, none of the top 10 most significant enrichment pathways upregulated by AAT were shared between the in vivo and ex vivo samples, making it difficult to determine whether key roles can be ascribed to any of the altered lncRNAs that may impact upon these specific processes. Previously, we reported on microRNAs that may affect specific pathways downregulated by AAT [11] and herein we propose that altered lncRNA expression may also contribute to the downregulation of genes involved in processes related to cell development and differentiation and morphogenesis. Further studies are required to explore this in more depth. 

There are limitations to this work including the small size of the study and the use of pooled samples. Furthermore, the lack of experimental validation studies is a problem due to no further patient material being available with which to perform validations; some of the very high fold changes observed on the array may be anomalous and validation studies to clarify this are an essential next step. Including non-AAT-deficient MM control samples in the study would have provided information about the normal lncRNA profile of monocytes. This would also have helped to determine the probable beneficial effect of AAT replacement therapy on the lncRNA profile of the monocytes. Notwithstanding these issues, we believe that reporting these observational data is worthwhile and will be of interest to AAT researchers and clinicians. To our knowledge, nothing has been reported on the role of lncRNAs in AAT deficiency, although their role in non-AAT deficiency COPD is becoming more widely studied. AAT deficiency is a genetic cause of COPD and is inherited in an autosomal co-dominant fashion. Consequently, *SERPINA1* MZ heterozygosity is a known risk factor for COPD in smokers [15]; the data presented here may be helpful to researchers studying cigarette smoke-induced COPD. In conclusion, this study shows an altered lncRNA expression profile in ZZ monocytes in response to AAT both in vivo and ex vivo. 

## 4. Methods

### 4.1. Study Population

AAT-deficient ZZ individuals receiving AAT augmentation therapy (n = 5) were recruited in this study as previously described [11]. ZZ individuals were receiving plasma-purified AAT from CSL Behring (Zemaira), administered intravenously at a dosage of 60 mg/kg body weight weekly; Day 0 refers to pre-AAT augmentation therapy whilst Day 2 refers to post-AAT augmentation therapy. All methods were carried out in accordance with relevant guidelines and regulations of the research ethics committee of Beaumont Hospital Dublin, who approved this study. Full informed consent was obtained from all subjects. 

### 4.2. Isolation, Culture and Treatment of Peripheral Blood Monocytes

Mononuclear cells were isolated using heparinized venous peripheral blood and treated with Lymphoprep (Axis Shield, Dundee, UK), for density gradient centrifugation. The mononuclear cell band was aspirated, washed with HBSS (Invitrogen, Dublin, Ireland) and monocytes were purified using the EasySep Human CD14 Selection Cocktail (StemCell Technologies, Vancouver, BC, Canada). The cells were cultured at 37 °C in 5% CO_2_ in the following media components from Life Technologies: RPMI 1640 containing 10% (*vol*/*vol*) fetal bovine serum plus 1% antibiotics (penicillin/streptomycin, Invitrogen). Treatment with exogenous AAT (Athens Research & Technology, Athens, GA, USA) at 27.5 µM for 4 h was performed to validate the effects of AAT replacement ex vivo.

### 4.3. Isolation of RNA, RNA Labeling and Array Hybridization 

RNA was isolated from purified monocytes with Tri Reagent (Sigma-Aldrich, Dublin, Ireland) as per the manufacturer’s instructions. The quality and the concentration of pooled RNA samples were monitored at absorbance ratios of A260/A280 and A260/A230 using a NanoDrop 8000 spectrophotometer. Expression profiling studies were performed on RNA by Arraystar, Inc. (Rockville, MD, USA). Briefly, labeled cRNAs were hybridized onto the Human LncRNA Array v3.0 (8 × 60 K, Arraystar) and after having washed the slides, the arrays were scanned by the Agilent Scanner G2505C. Approximately 30,600 lncRNAs and 26,109 coding transcripts can be detected using this microarray [11]. 

### 4.4. Data Analysis 

Agilent (Santa Clara, USA) Feature Extraction software (version 11.0.1.1) was used to analyze the acquired array images (carried out by Arraystar (Rockville, MD, USA) as a commercial service. Quantile normalization and subsequent data processing were performed using the GeneSpring GX v12.0 software package (Agilent Technologies, carried out by Arraystar, Rockville, MD, USA). After quantile normalization of the raw data, lncRNAs with flags in ≥1 out of all samples for Present or Marginal (i.e., “All Targets Value”) were chosen for further data analysis. Differentially expressed lncRNAs and mRNAs were identified through Fold Change filtering (> or <2.0) between two samples. Hierarchical Clustering was performed to show distinguishable expression patterns between samples. Pathway analysis and gene ontology (GO) analysis were applied to determine the potential roles of the differentially expressed mRNAs. 

## Figures and Tables

**Table 1:** The Top 10 up- and downregulated lncRNAs in AAT-deficient patients receiving AAT therapy versus those not receiving AAT therapy.

Probe Name	Fold Change	Log_2_ FC	Up-/Downregulated	Sequence Name	Gene Symbol
ASHGA5P037206	3809.9	11.9	Up	ENST00000430815	C21orf91-OT1
ASHGA5P031008	3229.1	11.7	Up	ENST00000448643	RP11-479J7.1
ASHGA5P056270	3169.5	11.6	Up	TCONS_00001566	XLOC_000905
ASHGA5P034590	2695.8	11.4	Up	ENST00000452073	AC092295.5
ASHGA5P015625	2468.78	11.3	Up	NR_051994	DIAPH3-AS1
ASHGA5P047974	2205.78	11.1	Up	NR_049725	NAV2-AS5
ASHGA5P058933	2088.8	11.03	Up	uc.92+	uc.92
ASHGA5P055648	2051.5	11.0	Up	ENST00000417460	AC003986.7
ASHGA5P029317	1351.3	10.4	Up	TCONS_00010873	XLOC_004700
ASHGA5P035595	1191.5	10.2	Up	uc001bgg.1	AK025975
ASHGA5P030089	−7931.1	−12.95	Down	ENST00000559861	RP11-265M18.2
ASHGA5P036057	−5954.6	−12.93	Down	TCONS_00026835	XLOC_013154
ASHGA5P039386	−5670.7	−12.5	Down	ENST00000509449	RP11-360F5.1
ASHGA5P017396	−1561.1	−10.6	Down	ENST00000441018	KCNMB2-IT1
ASHGA5P023626	−1283.9	−10.3	Down	HMlincRNA1110+	HMlincRNA1110
ASHGA5P031124	−1036.6	−10.0	Down	ENST00000569199	RP11-1437A8.6
ASHGA5P037608	−933.0	−9.86	Down	ENST00000430707	AP000347.4
ASHGA5P055474	−902.4	−9.82	Down	TCONS_00015528	XLOC_007235
ASHGA5P044777	−898.4	−9.81	Down	ENST00000518535	RP11-723D22.2
ASHGA5P021038	−836.7	−9.7	Down	ENST00000523781	CTB-17P3.4

**Table 2:** The Top 10 up- and downregulated lncRNAs in AAT-deficient monocytes treated ex vivo with AAT versus those not treated ex vivo with AAT.

Probe Name	Fold Change	Log_2_ FC	Up-/Downregulated	Sequence Name	Gene Symbol
ASHGA5P022322	7893.8	12.95	Up	ENST00000554529	RP11-14J7.6
ASHGA5P042735	7344.9	12.8	Up	ENST00000439207	RP11-568A7.2
ASHGA5P011052	6757.5	12.72	Up	ENST00000422819	CCDC162P
ASHGA5P029573	6669.8	12.70	Up	ENST00000557322	CTD-2002H8.2
ASHGA5P057456	4693.6	12.2	Up	TCONS_00017630	XLOC_008282
ASHGA5P035826	4089.4	12.0	Up	ENST00000439606	AC007254.3
ASHGA5P035237	3294.4	11.7	Up	ENST00000453478	AC068491.1
ASHGA5P027333	2953.9	11.6	Up	TCONS_00017353	XLOC_008198
ASHGA5P033331	2315.7	11.18	Up	ENST00000433856	AC003051.1
ASHGA5P058916	2272.5	11.15	Up	uc.268+	uc.268
ASHGA5P056269	−6780.7	−12.7	Down	TCONS_00001565	XLOC_000905
ASHGA5P030398	−6063.4	−12.6	Down	TCONS_00009189	XLOC_004205
ASHGA5P032563	−5563.0	−12.4	Down	ENST00000576014	RP11-166P13.4
ASHGA5P032930	−5105.5	−12.3	Down	ENST00000580225	AC004702.2
ASHGA5P045942	−3974.7	−11.96	Down	ENST00000433572	RP11-542K23.7
ASHGA5P050627	−3961.0	−11.95	Down	uc002tjp.2	DKFZp686E10196
ASHGA5P030018	−3461.2	−11.8	Down	NR_026891	FLJ10038
ASHGA5P027135	−2799.5	−11.5	Down	NR_038227	LOC100506451
ASHGA5P058034	−2775.1	−11.4	Down	TCONS_00025566	XLOC_012393
ASHGA5P051790	−2246.8	−11.1	Down	ENST00000461063	SOX2-OT

**Table 3:** LncRNAs upregulated in vivo and in ZZ monocytes treated ex vivo with AAT.

Sequence Name	Log_2_ FC In Vivo	Log_2_ FC Ex Vivo	Features
ENST00000452073	11.3964871	7.6158142	c/s 19, natural antisense
NR_049725	11.1070238	6.3263702	c/s 11, intronic antisense
uc.92+	11.0284561	1.7144114	c/s 2, intronic antisense

**Table 4:** LncRNAs upregulated ex vivo and in ZZ monocytes receiving augmentation therapy on Day 2.

Sequence Name	Log_2_ FC Ex Vivo	Log_2_ FC In Vivo	Features
ENST00000554529	12.9464952	8.2979554	c/s 14, intronic antisense
ENST00000439606	11.997679	4.0002684	c/s 2, intergenic
ENST00000453478	11.6858148	7.375152	c/s 2, intergenic
TCONS_00017353	11.5284172	4.525568	X c/s, intergenic
uc.268+	11.150079	6.470752	c/s 9, intronic antisense

## Data Availability

Data generated or analyzed during this study are included in this article and its Supplementary Information files and are also available on request from the corresponding author. The data are not publicly available due to General Data Protection Restrictions in the Republic of Ireland.

## Supplementary Materials

The following supporting information can be downloaded at: https://www.mdpi.com/article/10.3390/ncrna9010006/s1, Supplementary Dataset S1: All differentially expressed lncRNAs. Figure S1: Heat maps of lncRNA expression variation. Table S1: LncRNAs increased in the ex vivo AAT-treated ZZ monocytes and in the ZZ monocytes receiving AAT augmentation therapy in vivo (i.e., Day 2); Table S2: LlncRNAs decreased in the ex vivo AAT-treated ZZ monocytes and in the ZZ monocytes receiving AAT augmentation therapy in vivo (i.e., Day 2).
