# Supplementary material for: Long Non-Coding RNA Expression in Alpha-1 Antitrypsin Deficient Monocytes Pre- and Post-AAT Augmentation Therapy
Source: Noncoding RNA. 2023 Jan 10;9(1):6. doi: 10.3390/ncrna9010006 (PMC9844503; doi:10.3390/ncrna9010006)
Supplement: Supplementary file 1 [file ncrna-09-00006-s001.zip › ncrna-1983663-supplementary/ncrna-1983663-Figure S1.pdf]

A. B.

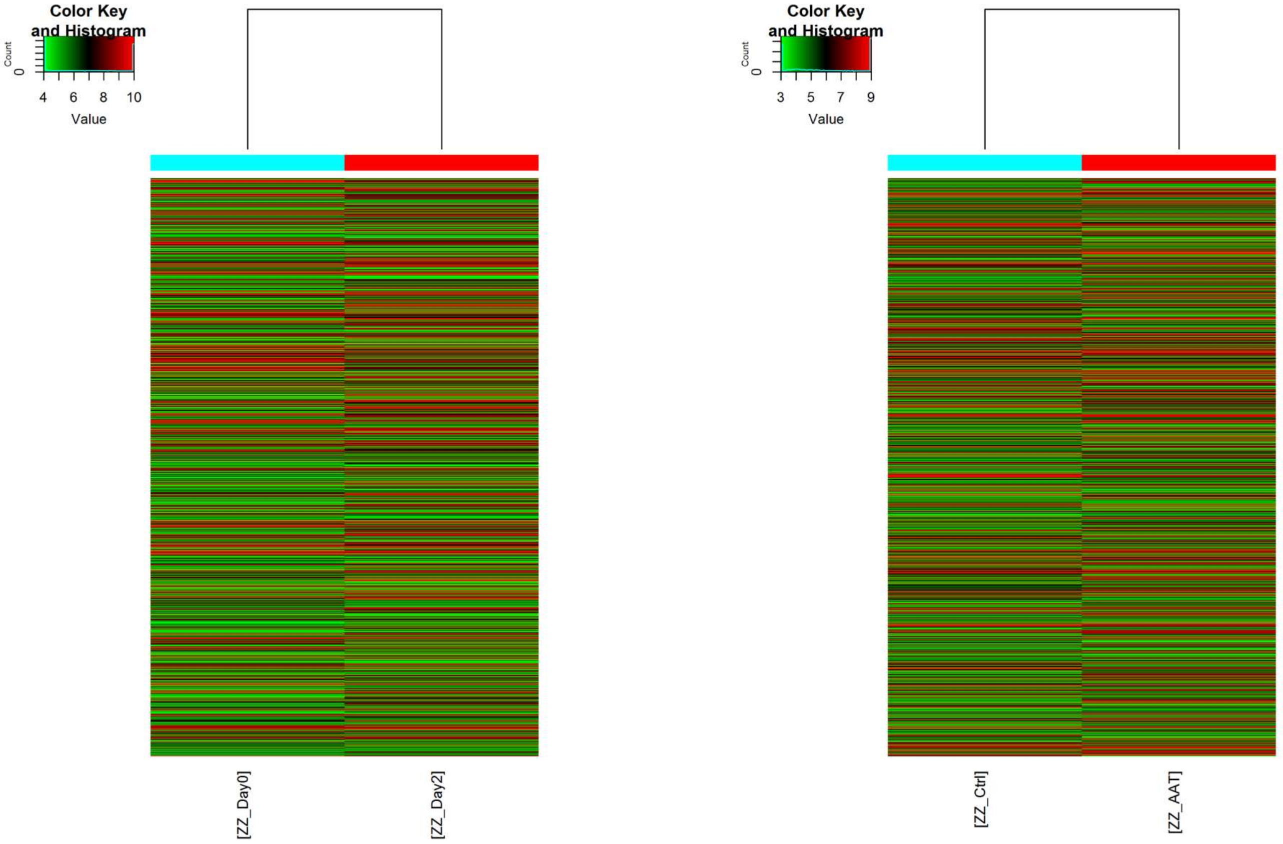

Supplementary Figure S1. Heat maps of lncRNA expression variation between (A) ZZ monocytes isolated 48 hours post AAT augmentation therapy (ZZ_Day 2) and ZZ monocytes from untreated individuals ZZ_(Day 0), and (B) ZZ monocytes treated ex vivo with 27.5 µM AAT for 4 hours (ZZ_AAT) and untreated control ZZ monocytes (ZZ_Ctrl).
